# Mapping the global endemicity and clinical burden of *Plasmodium vivax*, 2000–17: a spatial and temporal modelling study

**DOI:** 10.1016/S0140-6736(19)31096-7

**Published:** 2019-07-27

**Authors:** Katherine E Battle, Tim C D Lucas, Michele Nguyen, Rosalind E Howes, Anita K Nandi, Katherine A Twohig, Daniel A Pfeffer, Ewan Cameron, Puja C Rao, Daniel Casey, Harry S Gibson, Jennifer A Rozier, Ursula Dalrymple, Suzanne H Keddie, Emma L Collins, Joseph R Harris, Carlos A Guerra, Michael P Thorn, Donal Bisanzio, Nancy Fullman, Chantal K Huynh, Xie Kulikoff, Michael J Kutz, Alan D Lopez, Ali H Mokdad, Mohsen Naghavi, Grant Nguyen, Katya Anne Shackelford, Theo Vos, Haidong Wang, Stephen S Lim, Christopher J L Murray, Ric N Price, J Kevin Baird, David L Smith, Samir Bhatt, Daniel J Weiss, Simon I Hay, Peter W Gething

**Affiliations:** a Malaria Atlas Project, Big Data Institute, Li Ka Shing Centre for Health Information and Discovery, University of Oxford, Oxford, UK; b Menzies School of Health Research and Charles Darwin University, Darwin, NT, Australia; c Seattle & King County Public Health, Seattle, WA, USA; d Public Health England, Bristol, UK; e Medical Care Development International, Silver Spring, MD, USA; f Global Health Division, RTI International, Washington, DC, USA; g Epidemiology and Public Health Division, School of Medicine, University of Nottingham, Nottingham, UK; h Institute for Health Metrics and Evaluation, University of Washington, Seattle, WA, USA; i Eijkman-Oxford Clinical Rearch Unit, Jakarta, Indonesia; j Imperial College London, London, UK; k Centre for Tropical Medicine and Global Health, Nuffield Department of Medicine, University of Oxford, Oxford, UK

## Abstract

**Background:**

*Plasmodium vivax* exacts a significant toll on health worldwide, yet few efforts to date have quantified the extent and temporal trends of its global distribution. Given the challenges associated with the proper diagnosis and treatment of *P vivax*, national malaria programmes—particularly those pursuing malaria elimination strategies—require up to date assessments of *P vivax* endemicity and disease impact. This study presents the first global maps of *P vivax* clinical burden from 2000 to 2017.

**Methods:**

In this spatial and temporal modelling study, we adjusted routine malariometric surveillance data for known biases and used socioeconomic indicators to generate time series of the clinical burden of *P vivax*. These data informed Bayesian geospatial models, which produced fine-scale predictions of *P vivax* clinical incidence and infection prevalence over time. Within sub-Saharan Africa, where routine surveillance for *P vivax* is not standard practice, we combined predicted surfaces of *Plasmodium falciparum* with country-specific ratios of *P vivax* to *P falciparum*. These results were combined with surveillance-based outputs outside of Africa to generate global maps.

**Findings:**

We present the first high-resolution maps of *P vivax* burden. These results are combined with those for *P falciparum* (published separately) to form the malaria estimates for the Global Burden of Disease 2017 study. The burden of *P vivax* malaria decreased by 41·6%, from 24·5 million cases (95% uncertainty interval 22·5–27·0) in 2000 to 14·3 million cases (13·7–15·0) in 2017. The Americas had a reduction of 56·8% (47·6–67·0) in total cases since 2000, while South-East Asia recorded declines of 50·5% (50·3–50·6) and the Western Pacific regions recorded declines of 51·3% (48·0–55·4). Europe achieved zero *P vivax* cases during the study period. Nonetheless, rates of decline have stalled in the past five years for many countries, with particular increases noted in regions affected by political and economic instability.

**Interpretation:**

Our study highlights important spatial and temporal patterns in the clinical burden and prevalence of *P vivax*. Amid substantial progress worldwide, plateauing gains and areas of increased burden signal the potential for challenges that are greater than expected on the road to malaria elimination. These results support global monitoring systems and can inform the optimisation of diagnosis and treatment where *P vivax* has most impact.

**Funding:**

Bill & Melinda Gates Foundation and the Wellcome Trust.

## Introduction

Marked progress in malaria control has nearly halved deaths due to the disease since 2000 and reduced global incidence by more than 30%.^1^ Yet much of this progress was driven by a focus on *Plasmodium falciparum* malaria, the most prevalent and virulent malaria strain, which predominates in sub-Saharan Africa. To bring the global health community's ambition of eradicating malaria by 2040 closer to reality,^2^ it is crucial to gain a greater understanding of the other five *Plasmodium* species known to cause human malaria.^3^ *Plasmodium vivax* is the most geographically widespread species and second largest contributor to clinical (symptomatic) malaria worldwide, yet historically, its place on the international health agenda has remained low. Once viewed as a benign infection, *P vivax* malaria is now recognised as a cause of severe morbidity and mortality, resulting in a substantial negative effect on health worldwide.^4^, ^5^

Understanding the spatiotemporal distribution and clinical burden of *P vivax* is essential for effective control and elimination planning. *P vivax* often circulates in the peripheral blood at low, but transmissible, parasite densities. It also has the ability to form undetectable dormant liver stages (ie, hypnozoites), which can periodically awaken to cause recurrent infection and disease (relapse). Although 8-aminoquinoline drugs are effective against liver stage parasites, they can cause severe haemolytic anaemia in individuals with a glucose-6-phosphate dehydrogenase deficiency and are contraindicated in pregnant women and infants.^6^ Primaquine, the only widely available hypnozoiticide, is limited by poor adherence to the standard 14-day regimen and has reduced efficacy in patients with impaired cytochrome P-450 2D6 activity.^7^, ^8^, ^9^ The much-anticipated availability of tafenoquine will address some of these limitations but it remains a controlled therapy in the short-term, contingent on quantitative glucose-6-phosphate dehydrogenase screening, which is currently not widely available. Robust burden estimates can help identify areas where targeted use of such treatment should be a priority.

**Research in context**

**Evidence before this study**

*Plasmodium vivax* malaria is a harmful globally widespread disease, yet little work has been done to provide a comprehensive understanding of its morbidity worldwide. Previous assessments of clinical burden used case numbers reported to WHO adjusted by regional ratios of *P vivax* to *Plasmodium falciparum* infections or non-*falciparum* estimates from prevalence-based predictions. These approaches estimated 72 to 391 million incident cases per year. A 2012 study used survey-based infection prevalence estimates from microscopy or rapid diagnostic tests to generate a global map of *P vivax* malaria prevalence for 2010. However, this survey-based approach might be less well suited for mapping *P vivax* worldwide because of the parasite's biology. *P vivax* is often present in peripheral blood at subpatent densities and infections include a dormant liver stage invisible to current diagnostic methods. Further, population-based surveys that collect malariometric data are far less common outside of Africa—areas where *P vivax* predominates. Routine surveillance reports, which are increasingly available from ministries of health, provide longitudinal data with a greater likelihood of capturing clinical cases resulting from *P vivax* infections (primary or relapsing) than cross-sectional surveys.

**Added value of this study**

In this study we provide the first, to the best of our knowledge, formal quantification of *P vivax* malaria incidence and infection prevalence over time and across all endemic countries. We substantially expanded data sources included in the overall model framework relative to previous efforts, drawing on national level case reports, nearly 10 000 administrative unit-years of reported surveillance data, and over 14 000 prevalence survey locations. We sought to address known biases in the surveillance data, drawing from algorithms developed by WHO to account for reporting, presumptive diagnoses, and care-seeking behaviours. By mapping *P vivax* disease burden at a resolution of 5 × 5 km, our study enables comparison with other malaria parasites and a more in-depth examination of where the largest obstacles to overall malaria elimination currently occur.

**Implications of all the available evidence**

Globally, the *P vivax* malaria burden has decreased by 42% since 2000, falling from 24·5 million cases in 2000 to 14·3 million cases in 2017. Malaria burden peaked around 2005 and since then the burden of *P vivax* malaria nearly halved (−46%). During that time, large areas in Asia and the Americas achieved full parasite elimination or unstable transmission (<0·1 case per 1000 person-years observed), representing key milestones toward elimination. Nonetheless, progress in controlling *P vivax* has stalled across endemic regions in the past 5 years, and in areas of the Middle East and South America, disease burden has increased since 2013. The inability to quantify rates of *P vivax* malaria estimates for much of sub-Saharan Africa remains a challenge, emphasising the need to improve species-specific surveillance in the region. These global maps and corresponding disease estimates serve as essential inputs for malaria control and elimination programmes, pinpointing where past efforts have been successful and where heightened intervention is required to bend *P vivax* trajectories toward zero.

Previous efforts to quantify the burden of *P vivax* malaria were derived from *P falciparum* or total malaria estimates, and varied widely. An estimate of 72–80 million cases was published in 2001^10^ based on case numbers and regional ratios of *P vivax* to *P falciparum* infections reported to WHO. In 2007, Price and colleagues predicted 132–391 million cases based on non-falciparum malaria prevalence estimates generated by Hay and colleagues.^11^, ^12^, ^13^ In 2013, WHO began including *P vivax* case estimates in the World Malaria Report and then reported 11·9–22·0 million *P vivax* clinical cases for 2012.^14^ Establishing where precisely the true burden of *P vivax* malaria lies, with respect to the wide range of these conflicting estimates (12–390 million), has long been identified as a research priority.^10^, ^12^, ^15^

The aim of this analysis was to quantify and map clinical *P vivax* malaria cases and the prevalence of patent blood-stage infections in all *P vivax* endemic areas from 2000 to 2017. Building on previous analyses that mapped endemicity using cross-sectional survey data for infection prevalence,^16^ substantial methodological improvements allowed the wealth of information collected by routine surveillance activities to be used. As a result of these developments, *P vivax* was added to the Global Burden of Disease (GBD) cause list. The findings presented here, together with parallel work on *P falciparum*,^17^ informed the overall malaria estimates for the GBD 2017 study.^18^ Here, results are presented at a fine scale (5 × 5 km) and for *P vivax* alone. The maps and trends provide an evidence-base to track past and future progress towards national and regional milestones and to identify areas in which improved diagnosis and treatment efforts should be targeted on the path to malaria elimination.

## Methods

### Overview

Separate models were developed for *P vivax* clinical incidence and infection prevalence (parasite rate) within and outside Africa, reflecting the nature and quality of available data. A surveillance-based approach was primarily used for locations outside Africa, with clinical incidence modelled from routine surveillance reports obtained from country programmes (Malaria Atlas Project database) and the World Malaria Report. Time-series models generated national case counts that informed pixel-level clinical incidence predictions within endemic areas. A model of the relationship between clinical incidence and *P vivax* parasite rate converted fine-scale incidence into estimates of prevalence.^19^ Within Africa, data were not collected as part of routine surveillance or cross-sectional surveys across most of the continent due to the dominance of the host Duffy-negative blood group that was thought to prevent endemic transmission of the parasite.^20^, ^21^ Estimates for *P vivax* in Africa therefore started with fine-scale estimates of *P falciparum* parasite rate derived from household survey data^22^, ^23^ with a cartographic modelling approach.^24^ *P falciparum* incidence was modelled from these *P falciparum* parasite rate estimates,^25^ and then converted to *P vivax*-specific incidence using reported country-specific and year-specific ratios of *P vivax* to *P falciparum*.^26^ Schematic diagrams of the analyses used are provided in the appendix (pp 61–62).

This analysis adheres to Guidelines for Accurate and Transparent Health Estimates Reporting standards (GATHER; appendix p 63). Statistical code is available through an online repository. Analyses were done with R version 3.4.4 or later.

### Data assembly

For locations with subnational surveillance data (ie, the Americas, Europe, Asia, and Eritrea and Djibouti in Africa), data for annual parasite incidence^27^ were gathered from publicly available routine passive surveillance reports, peer-reviewed journals, and online portals (eg, ministry of health websites). Data were extracted to the highest spatial resolution available, in the form of annual parasite incidence or case counts (appendix pp 4–6). Parasite rate point data from the Malaria Atlas Project database were used to model rates within Africa and to inform the surveillance-based models outside Africa. The methods used to develop, maintain, and routinely update this database are described elsewhere.^16^, ^24^

Between 2000 and 2017, routine surveillance data were available from 40 countries, comprising 9867 administrative units (appendix p 18). *P vivax* parasite rate surveys informed both the *P vivax* incidence downscaling model and the incidence to prevalence conversion. The Malaria Atlas Project database contained 14 412 *P vivax* parasite rate records beginning 1985 (appendix pp 26–27).

### Surveillance report adjustments

Raw counts of passively detected cases aggregated through routine surveillance were adjusted to account for annual reporting rates, presumptive diagnoses, and treatment-seeking behaviours (appendix pp 7–9), consistent with algorithms applied to reported case data by WHO for its annual World Malaria Report.^28^ Reports from countries in elimination stage^29^ were not adjusted as they were considered to have robust health systems able to capture and report all local cases.

Subnational estimates of reporting completeness (ie, proportion of expected reports actually received from health facilities) were available for a few countries from online reports or shared directly with the Malaria Atlas Project. Otherwise, national counts were taken from the corresponding year in the 2017 World Malaria Report.^26^ When unconfirmed (presumptively diagnosed) cases were reported, these were adjusted by the slide positivity rate of confirmed cases from the same year for a particular country. Reports were also adjusted for the proportion of fever cases that seek treatment, on the basis of modelled household data from demographic and health surveys and malaria indicator surveys.^23^ These treatment-seeking models were informed by socioeconomic indicators from the World Bank, and gap-filled for missing years using a Gaussian process regression. Although rates were calculated from child-specific data, it was assumed that treatment-seeking patterns would be similar across age groups.^26^, ^30^

### Estimating incidence within Africa

There is now substantial evidence that *P vivax* is endemic throughout Africa,^20^, ^31^ though local surveillance for this parasite remains rare. Only six countries reported more than 0% of all malaria cases as attributable to *P vivax*: Djibouti, Eritrea, Ethiopia, Madagascar, Somalia, and Sudan.^29^ Countries around the Horn of Africa and Madagascar have a lower prevalence of the Duffy-negative phenotype^32^ and it is more widely accepted that these regions can sustain endemic transmission of *P vivax*. Djibouti and Eritrea were modelled using surveillance-based methods because subnational case data were available. Elsewhere, the percentage of cases attributable to *P vivax* (relative to *P falciparum*) was assembled from species-specific surveillance reports for the closest year match in the World Malaria Report. These proportions were used to transform pixel-level *P falciparum* predictions into *P vivax* incidence.

Prevalence and incidence maps of *P falciparum* for 2000–17 were modelled from cross-sectional data using the geostatistical framework described by Bhatt and colleagues.^24^ A fully Bayesian latent Gaussian model predicted *P falciparum* parasite rate from the survey point data, informed by temporally dynamic environmental covariates and modelled intervention coverage surfaces. *P falciparum* parasite rate estimates at 5 × 5 km resolution were converted to *P falciparum* incidence using the modelled relationship previously described.^25^ In contrast to the 1–99 year age range modelled for *P vivax*, the *P falciparum* model estimated parasite rate and clinical incidence in children aged 2–10 years. Outputs were standardised to all ages using established age-standardisation relationships^16^, ^33^ before conversion to *P vivax*.

### Estimating incidence outside Africa

National World Malaria Report case count data were adjusted as described above and used to derive case estimates and incidence from 2000 to 2017 (based on GBD national population estimates).^34^ Time-series models were used to estimate incidence for country-years when data were not available. Log-incidences were modelled using country-specific intercepts and linear relationships with gap-filled, open-access World Bank covariates, including annual gross domestic product growth and relative government health expenditure.^35^ Short-term and long-term moving averages were applied to account for residual temporal variation. To share information between countries, long-term moving averages were applied regionally or subregionally (appendix p 20).

In addition to national models, Brazil, China, India, Indonesia, Iran, Mexico, and South Africa were modelled subnationally at the first administrative unit for consistency with the GBD study.^18^ Because World Bank covariate data were only available nationally, analogous subnational variables provided by the GBD study were used in a similar modelling framework to the national models, with the additional constraint that subnational counts summed to national values (appendix p 20).

Time-series outputs provided national (or subnational) incidences for each country-year. To generate pixel outputs from administrative-unit level incidence, an ecological downscaling Bayesian model was used,^36^, ^37^ linking each administrative unit's cases to a latent fine-scale map by an appropriate areal integral in the likelihood function. The fine-scale structure was modelled within a machine-learning approach by a Gaussian random field informed by environmental and sociodemographic covariates and point observations of *P vivax* parasite rate from available cross-sectional surveys. Bootstrapping was used to obtain 100 samples that characterised the uncertainty in the model, summarised as pixel-level SD estimates and upper or lower incidence bounds (appendix pp 25–55). All model predictions were made within the geographical limits of *P vivax* transmission, defined by environmental layers of temperature and aridity, together with surveillance reports detailing subnational areas within endemic countries known to be *P vivax*-free.^38^ All pixel level outputs are scaled to respect the national totals generated from time-series modelling, thereby introducing the potential for distinct border effects to be observed in mapped outputs.

### Converting clinical incidence to infection prevalence

Global *P vivax* parasite rate maps were modelled directly from pixel-level estimates of *P vivax* clinical incidence, informed by two input datasets consisting of matched *P vivax* parasite rate and incidence measurements. The first was a previously published dataset of paired measures of *P vivax* clinical incidence (from active case detection studies), matched spatially and temporally to measures of *P vivax* parasite rate.^39^ The second dataset was derived by geographically matching the cross-sectional *P vivax* parasite rate points from the Malaria Atlas Project database to incidence reports from the subnational surveillance data. Data from both sets were grouped by relapse zone.^40^ The periodicity of relapse varies geographically, thus influencing the number of clinical cases expected to arise from a single infection identified during a community survey. We fitted a Bayesian mixed-effects logistic regression model with a random slope and intercept for incidence in each relapse zone. This method allowed the relationship between prevalence and incidence to be estimated separately for each zone, while enabling areas with little data to borrow strength from the whole dataset (appendix pp 6–7).

### Role of the funding source

The funder of the study had no role in study design, data collection, data analysis, data interpretation, or writing the report. All authors had full access to the data in the study and had final responsibility for the decision to submit for publication.

## Results

**Figure 1:**
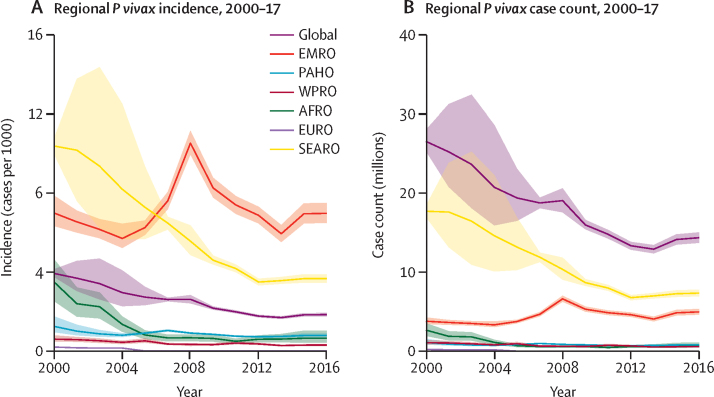
Temporal trends in *Plasmodium vivax* incidence and case counts from 2000 to 2017 The lines represent the temporal trends of incidence (A) and clinical case counts (B) of each WHO region. The shaded areas represent the 95% uncertainty intervals of these estimates. EMRO=Eastern Mediterranean Regional Office. PAHO=Pan American Health Organization. WPRO=Regional Office for the Western Pacific. AFRO=Regional Office for Africa. EURO=Regional Office for Europe. SEARO=Regional Office for South-East Asia.

**Figure 2:**
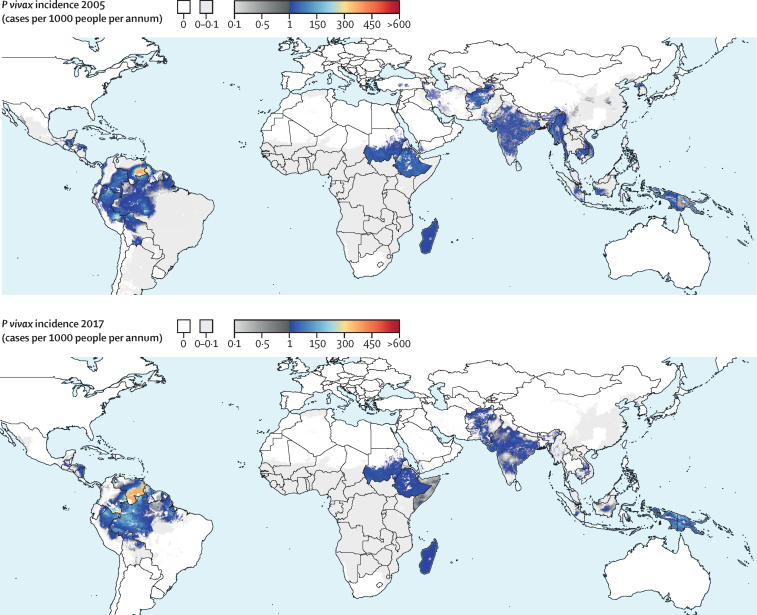
Predicted incidence of *Plasmodium vivax* malaria in 2005 and 2017 Incidence in cases per 1000 people per year are shown on a spectrum of white (zero incidence) to dark grey (1 case per 1000) and then blue to red (>1 case per 1000 to >600 cases per 1000) for the years 2005 (top panel) and 2017 (bottom).

**Figure 3:**
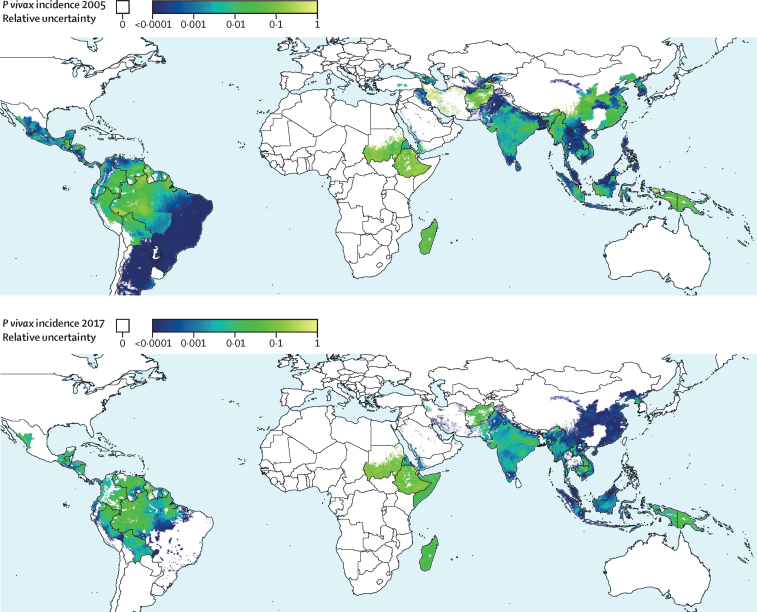
Relative uncertainty of *Plasmodium vivax* incidence pixel-level allocation The relative uncertainty values for 2005 (top) and 2017 (bottom), as calculated by the SD divided by the square root of the mean are shown on a spectrum of blue (most certain) to yellow (least certain). These uncertainty values relate to distribution of cases rather than the certainty of the case counts themselves.

**Figure 4:**
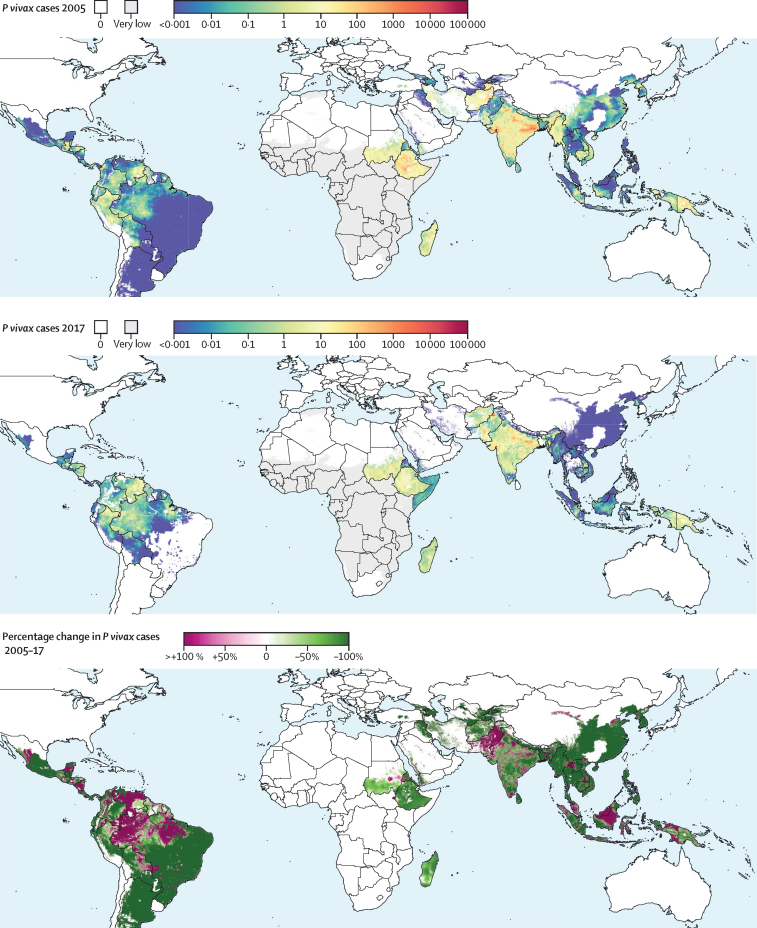
Predicted *Plasmodium vivax* clinical cases and change from 2005 and 2017 The numbers of cases predicted to occur in each 5 × 5 km pixel are shown on a spectrum of blue to red for the years 2005 (top panel) and 2017 (middle). Areas where *P vivax* is known to be endemic, but there was not sufficient information to generate a prediction are shown in light grey. The bottom panel shows change calculated by the value for 2005 minus 2017 divided by the 2005 value and multiplied by 100, such that a decrease is shown on a scale of white to green and an increase from white to pink. The darkest green areas have seen a 100% or greater decrease in *P vivax* cases from 2005 to 2017, while the darkest pink areas show a 100% or greater increase in cases.

**Figure 5:**
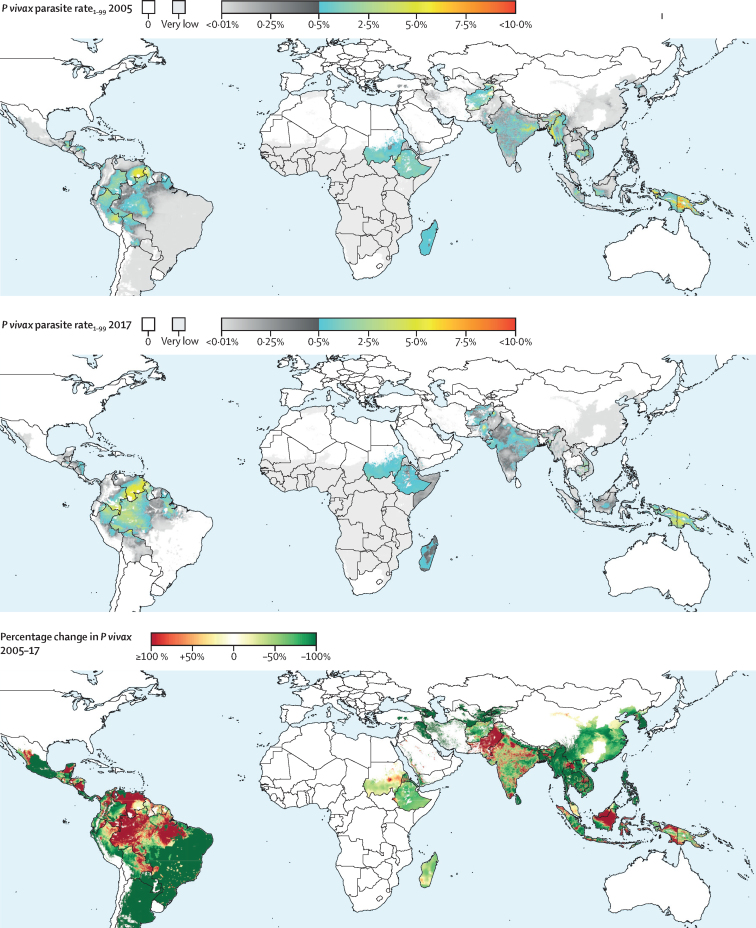
Predicted *Plasmodium vivax parasite rate* and change 2005 and 2017 The prevalence in ages 1 to 99 years predicted to occur in each 5 × 5 km pixel are shown on a spectrum of light blue to red for the years 2005 (top panel) and 2017 (middle). Areas where *P vivax* is known to be endemic, but there was not sufficient information to generate a prediction are shown in light grey. The bottom panel shows change calculated by the value for 2005 minus 2017 divided by the 2005 value and multiplied by 100, such that a decrease is shown on a scale of white to green and an increase from white to red. The darkest green areas have seen a ≥100% decrease in prevalence from 2005 to 2017, while the darkest red areas show a ≥100% increase in prevalence.

Globally, malaria incidence peaked between 2002 and 2005 (figure 1A). Global maps for incidence (figure 2) and its uncertainty (figure 3), clinical case counts (figure 4), and *P vivax* parasite rate (figure 5) for the years 2005 (the peak year for malaria cases globally) and 2017 show areas in which progress towards *P vivax* malaria has been achieved, stagnated, and in a few instances, reversed.

The global population living within the limits of *P vivax* transmission was 3·3 billion in 2017. This increase from 2·9 billion in 2000—despite 16 (15%) of 104 of the endemic countries eliminating transmission by 2017—was attributed to population growth (appendix p 58). In 2000, 29% (1·4 billion of 4·7 billion) of the population of *P vivax* endemic countries was at risk of stable transmission (areas with a predicted incidence ≥0·1 cases per 1000 per annum), decreasing to 27% (1·5 billion of 5·7 billion) in 2017 (appendix p 58).

*P vivax* malaria cases fell 41·6% (95% uncertainty interval [UI] 39·3–44·6) between 2000 and 2017 and 45·9% (45·9–46·3) between 2005 and 2017, from 24·5 million (22·5–27·0) in 2000 and 26·4 million (25·2–27·9) in 2005 to 14·3 million (13·7–15·0) in 2017 (figure 1B, table 1). South-East Asia drove this overall change. From 2005, cases in South-East Asia decreased by 58·5% (95% UI 57·7–59·2) with 10·3 million (10·0–10·8) fewer cases by 2017. This region also had the highest proportion of the global burden: 60·3% (14·8 million of 24·5 million) of all cases in 2000, 66·7% (17·6 million of 26·4 million) in 2005, and 51·2% (7·3 million of 14·3 million) in 2017. Other pronounced declines were in the Americas, which achieved 56·8% (95% UI 47·6–67·0) case reductions, the WHO African region, which achieved 79·7% (76·4–80·0) case reductions, and the WHO European region, which achieved 100% case reductions between 2000 and 2017. In the Eastern Mediterranean region, Djibouti and politically unstable countries such as Pakistan and Yemen contributed to a rise in absolute burden and incidence. Approximately three-quarters of the global burden of *P vivax* was consistently attributable to India, Pakistan, and Ethiopia: 72·3% in 2000 (India 12·5 million [95% UI 11·7–13·3], Pakistan 2·1 million [1·9–2·3], and Ethiopia 3·2 million [1·8–5·2]) and up to 80·3% in 2005 (India 16·4 million [15·7–17·2], Pakistan 2·3 million [2·1–2·5], Ethiopia 2·5 million [1·8–3·3]) and 79·5% in 2017 (India 6·8 million [6·4–7·2], Pakistan 4·0 million [3·8–4·3], Ethiopia 0·6 million [0·4–1·0]).

**Table 1:** Estimated case numbers in 2000, 2005, and 2017 for *Plasmodium vivax* in millions

	**Clinical burden 2000**	**Clinical burden 2005**	**Clinical burden 2017**	**Change from 2000 to 2017**	**Percentage change from 2000 to 2017**	**Change from 2005 to 2017**	**Percentage change from 2005 to 2017**
Regional Office for Africa	3·31 (1·86 to 5·40)	2·65 (1·92 to 3·52)	0·67 (0·44 to 1·08)	−2·64 (−1·42 to −4·32)	−79·62 (−76·20 to −80·06)	−1·98 (−1·48 to −2·44)	−74·54% (−76·87 to −69·45)
Eastern Mediterranean Regional Office	3·30 (3·00 to 3·64)	3·77 (3·41 to 4·24)	4·95 (4·60 to 5·32)	1·65 (1·61 to 1·68)	49·97 (53·85 to 46·01)	1·18 (1·18 to 1·08)	31·17%(34·65 to 25·38)
Regional Office for Europe	0·09 (0·07 to 0·12)	0·02 (0·02 to 0·02)	0·00 (0 to 0)	−0·09 (−0·07 to −0·12)	−100 (−100 to −100)	−0·21 (−0·21 to −0·21)	−100·00% (−100 to −100)
Pan American Health Organization	1·83 (1·33 to 2·70)	1·09 (0·08 to 1·54)	0·79 (0·70 to 0·89)	−1·04 (−0·63 to −1·81)	−56·82 (−47·64 to −67·00)	−0·31 (−0·14 to −0·64)	−27·99% (−16·64 to −42·02)
Regional Office for South-East Asia	14·78 (13·99 to 15·61)	17·63 (16·91 to 18·32)	7·31 (6·90 to 7·75)	−7·46 (−7·08 to −7·86)	−50·50 (−50·64 to −50·33)	−10·32 (−10·00 to −10·57)	−58·52% (−59·17 to −57·68)
Regional Office for the Western Pacific	1·18 (0·94 to 1·51)	1·06 (0·84 to 1·40)	0·57 (0·49 to 0·67)	−0·60 (−0·45 to −0·83)	−51·27 (−48·45 to −55·43)	−0·49 (−0·35 to −0·73)	−46·02% (−41·86 to −52·02)
Total	24·49 (22·51 to 27·00)	26·43 (25·23 to 27·88)	14·30 (13·65 to 14·97)	−10·19 (−8·86 to −12·04)	−41·61 (−39·35 to −44·58)	−12·13 (−11·58 to −12·91)	−45·90% (−45·88 to −46·32)

Data are the estimated case numbers in 2000, 2005, and 2017 reported by WHO region along with the total and percentage change over that time (95% uncertainty interval).

At the start of the study period in 2000, incidence was highest in South-East Asia, but from 2010 was eclipsed by rates in the Eastern Mediterranean region (figure 1A). The highest proportions (>100 cases per 1000 total population) at the start of the study period were in Melanesia: Papua New Guinea, Solomon Islands, and Vanuatu (Figure 2, Figure 3, Figure 4). By 2017, the ten countries with the highest proportions (ranging from 96·9 to 3·5 cases per 1000) were Afghanistan, Ethiopia, Guyana, India, Pakistan, Papua New Guinea, Solomon Islands, Sudan, Vanuatu, and Venezuela.

The relative change in case counts from 2005 to 2017 is shown in figure 4. Considerable progress is shown in decreasing case burden, but apart from the European region, this is punctuated by substantial increases. The most notable increases were in parts of the Americas and Asia. Changes in incidence and burden also reveal that as burden declined, the proportion of cases attributable to *P vivax* increased (table 2). Regions with the lowest overall burdens had higher ratios of *P vivax* (Europe and the Americas) relative to areas with consistently high burdens (South-East Asia).

**Table 2:** Change in proportion of cases attributable to *P vivax* from 2000 to 2017 by region

	**Clinical***Plasmodium vivax***burden**		**Clinical***Plasmodium falciparum***burden**		**Mean proportion of cases due to***P vivax*		**Change in***P vivax***proportion**
	2000	2017	2000	2017	2000	2017	
Eastern Mediterranean Regional Office	3·30	4·95	6·62	4·64	0·33	0·52	55·10%
Regional Office for Europe	0·09	0·00	0·00	0·00	0·96	NA	NA
Pan American Health Organization	1·83	0·79	1·15	0·41	0·61	0·66	7·32%
Regional Office for South-East Asia	14·78	7·31	16·12	7·82	0·48	0·48	1·02%
Regional Office for the Western Pacific	1·18	0·57	2·59	0·87	0·31	0·40	26·81%

The predicted case count estimates (in millions) for *P vivax* malaria are shown alongside *P falciparum*^17^ along with the percentage change in the proportion of cases due to *P vivax*. Regional Office for Africa was excluded due to insufficient evidence to quantify the species ratios in the region. NA=not applicable.

Uncertainty in the aggregate case estimates (figure 1, table 1) closely reflected data availability, whereas in the pixel-level predictions from downscaling areal incidence (figure 3), uncertainty was driven both by error in the input data and in the model used to disaggregate cases across space. Uncertainty in the maps must therefore be interpreted distinctly from the credible interval ranges of the case estimates themselves. Maps of the 95% uncertainty interval ranges are shown in the appendix (pp 58–59).

Pixel-level maps of all-age *P vivax* parasite rate and change from 2005 to 2017 are shown in figure 5. This metric reflects the prevalence of blood-stage *P vivax* infections detectable by microscopy. During the study period, many regions saw considerable reductions, with only isolated areas, such as Papua New Guinea, showing rates near or above 10% in 2017. The greatest reductions in parasite prevalence were across large parts of Mexico and Brazil, the Indian subcontinent, China, the Greater Mekong Subregion, and Indonesia. Conversely, areas of concern where prevalence increased, included focal zones within Pakistan and western India, and Venezuela, the highest prevalence region in the Americas in 2017, where prevalence has risen since 2012.

All maps, along with regional and national estimates, are freely available through the Malaria Atlas Project website.

## Discussion

The spatially and temporally rich dataset of *P vivax* case reports and prevalence measures assembled here informed the first ever maps of *P vivax* clinical incidence and *P vivax* parasite rate over time. The resulting maps and case summaries show impressive gains in control since the turn of the century, but also stagnating progress in the past 5 years and even increases in some areas. The biology of *P vivax* means that the clinical case estimates generated here represent only a proportion of the total parasite reservoir, which includes a large proportion of asymptomatic infections.^41^ Similarly, *P vivax* parasite rate maps based on detection using microscopy or rapid diagnostic tests (RDT) cannot represent the prevalence of low-density blood or liver-stage infections caused by *P vivax*. Nonetheless, because methods were consistent across regions, malaria control programmes might use these outputs to identify priority areas for control. Furthermore, as these analyses will be updated annually, the results serve as a baseline against which to assess future progress.

Overall incidence has declined in most regions since 2000. *P vivax* was eliminated from the European region during the study period and there were elimination successes elsewhere such as Paraguay and Sri Lanka. Other countries in South-East Asia, the Americas, and the Western Pacific are approaching elimination, with reported reductions in incidence of over 99% from 2000–17 in Belize, Bhutan, China, Ecuador, and Timor Leste. We found that as incidence declined, the proportion of malaria attributable to *P vivax* increased—both at regional and country scales. For instance, 65% of the approximate 50 000 malaria cases in China in 2000 were attributable to *P vivax,* whereas by 2017 the handful of remaining local cases were all *P vivax*. In Thailand, 42% of cases were *P vivax* in 2000 compared with 59% in 2017 following a 95% reduction in incidence. Although this pattern was not universal, the increasing proportions of *P vivax* in countries with declining transmission is a reminder of the need for strategies explicit to *P vivax* in elimination plans.^3^

Areas where the case burden increased during the study period serve as an urgent call to action on the part of policy makers for targeted funding and control strategies. Djibouti, Pakistan, and Venezuela were all notable in showing increased burden rather than declines as seen in neighbouring countries. Several more countries, however, had increases since 2013. Reversed progress in Afghanistan, Somalia, and Yemen emphasise the damaging impact of political and civil instability on the functionality of malaria control efforts. In such situations, reinforced external support to affected and neighbouring countries (Ecuador has also seen increased case incidence since 2013) is essential to avoid further losses.

An important strength of this work was the methodological developments that enabled a move away from reliance on cross-sectional prevalence data alone. *P vivax* is poorly suited to detection in cross-sectional surveys because of intrinsically low-density parasitaemia in the peripheral blood and hypnozoite forms in the liver, limiting the proportion of infections identifiable by standard field diagnostics. The shift towards routine surveillance data allowed greater geographical coverage of the raw data and provided a malaria metric more closely aligned with the final desired output indicators. These routine data are not without biases, however. Accurately accounting for treatment-seeking behaviours, presumptively diagnosed cases, and reporting completeness presented several key limitations. For example, due to national survey study design, treatment seeking rates were derived from children younger than 5 years with any cause of fever—not explicitly malaria-attributable. Although fever is an appropriate proxy for malaria in some high-endemicity contexts,^42^ in settings nearing elimination, treatment-seeking for fever might be far lower than for malaria, leading to possible overestimates of the malaria burden. Additionally, lower-endemicity settings tend to be wealthier, with health systems more reliably detecting and reporting cases. Overall, adjusting for known biases highlighted persistent gaps in malaria data and the challenges inherent to monitoring *P vivax* malaria.

Our adjustments did not account for important shifts in diagnostic practices over the study period. For instance, increased accessibility to diagnosis through the widespread roll out of RDTs will have increased numbers of confirmed cases, while the use of species-specific RDTs might have reduced microscopy-associated risks of misdiagnosis. The sensitivity of *P vivax* diagnosis is reduced by characteristically low peripheral parasitaemias, but also by the sequestration of the parasite in the spleen and bone-marrow, rendering it undetectable by microscopy or RDT.^43^ Thus, our *P vivax* parasite rate maps represent an unknown proportion of all actual infections present in endemic populations.

Reflecting the routine surveillance data, the case estimates reported here do not differentiate between primary infections and relapses. In some contexts, relapses might represent 50–80% of overall *P vivax* cases.^40^, ^44^ To account for the varying effect of relapse, we used separate incidence-prevalence relationships for geographical areas with unique relapse frequency phenotypes.^40^ Understanding relapse frequency, however, is important because the greatest morbidity and mortality due to *P vivax* arises from this cumulative burden of repeated bouts of infection.^44^ Downstream burden analyses such as quantifying levels of disability-adjusted life-years might therefore benefit from this distinction, even though the total case incidence modelled here will not be affected by whether clinical episodes are primary infections or relapses.

A final limitation was that estimates could not be quantified for much of Africa because of a scarcity of data. Recent research has documented *P vivax* endemicity throughout the continent,^20^ albeit at low levels outside the Horn and parts of east Africa. In much of Africa, it is not routine to test or report non-*falciparum* species. Therefore, incidence and case numbers could only be estimated for Djibouti, Eritrea, Ethiopia, Madagascar, Somalia, and Sudan, where species-specific cases were reported and a *P vivax* ratio could be applied to *P falciparum* predictions. Djibouti and Eritrea had subnational information allowing estimates to be generated in the same way as countries outside of Africa. For the remaining African countries, incidence was given a uniform classification of unstable (<0·1 cases per 1000 per annum).

It is necessary for us to emphasise that the biases inherent in the input data and the challenges in generating predictions where data are missing means that the maps and outputs shown here are model summaries and approximations of the truth. Uncertainties in both the data and model predictions are carried through to the outputs and these communicated in additional summary figures (figure 3; appendix pp 58–60), which are important to consult when assessing the mapped predictions. The resolution of the data available for analysis (to inform both the time-series and subnational trends) carries important implications for the model's predictive capacity. It is hoped that future iterations of this work will allow predictions in these areas of uncertainty to be refined.

The near halving of *P vivax* burden globally since 2005 mirrors declines in *P falciparum*,^17^ illustrating the progress made by the global health community and international funding institutions in combating malaria. The danger associated with such successes, however, is the onset of complacency as the contribution to morbidity and mortality of malaria declines relative to other diseases. To avoid resurgences, the international focus and funding directed towards driving down malaria cannot wane. Although *P falciparum* rightly receives more attention because of its higher burden, *P vivax* presents distinct challenges to control and elimination. It is harder to detect, and patients who do not receive radical cure are susceptible to relapses and potentially severe disease and death. The results presented here contribute to our understanding of global *P vivax* epidemiology, characterising areas of persistent transmission and incomplete evidence. These findings emphasise the need for diagnostic practices sensitive to *P vivax* throughout the malaria-endemic world, alongside increased access to effective radical cure.

## Data sharing

All pixel-level and administrative-level summaries are available forvisualisation and download at https://map.ox.ac.uk/malaria-burden.
